# BiFFN: Bi-Frequency Guided Feature Fusion Network for Visible–Infrared Person Re-Identification

**DOI:** 10.3390/s25051298

**Published:** 2025-02-20

**Authors:** Xingyu Cao, Pengxin Ding, Jie Li, Mei Chen

**Affiliations:** School of Computer Science, Chengdu University of Information Technology, Chengdu 610225, China; xingyu200624@gmail.com (X.C.); dpx@cuit.edu.cn (P.D.); chenmei@cuit.edu.cn (M.C.)

**Keywords:** VI-ReID, frequency domain analysis, feature fusion, modality gap reduction

## Abstract

Visible–infrared person re-identification (VI-ReID) aims to minimize the modality gaps of pedestrian images across different modalities. Existing methods primarily focus on extracting cross-modality features from the spatial domain, which often limits the comprehensive extraction of useful information. Compared with conventional approaches that either focus on single-frequency components or employ simple multi-branch fusion strategies, our method fundamentally addresses the modality discrepancy through systematic frequency-space co-learning. To address this limitation, we propose a novel bi-frequency feature fusion network (BiFFN) that effectively extracts and fuses features from both high- and low-frequency domains and spatial domain features to reduce modality gaps. The network introduces a frequency-spatial enhancement (FSE) module to enhance feature representation across both domains. Additionally, the deep frequency mining (DFM) module optimizes cross-modality information utilization by leveraging distinct features of high- and low-frequency features. The cross-frequency fusion (CFF) module further aligns low-frequency features and fuses them with high-frequency features to generate middle features that incorporate critical information from each modality. To refine the distribution of identity features in the common space, we develop a unified modality center (UMC) loss, which promotes a more balanced inter-modality distribution while preserving discriminative identity information. Extensive experiments demonstrate that the proposed BiFFN achieves state-of-the-art performance in VI-ReID. Specifically, our method achieved a Rank-1 accuracy of 77.5% and an mAP of 75.9% on the SYSU-MM01 dataset under the all-search mode. Additionally, it achieved a Rank-1 accuracy of 58.5% and an mAP of 63.7% on the LLCM dataset under the IR-VIS mode. These improvements verify that our model, with the integration of feature fusion and the incorporation of frequency domains, significantly reduces modality gaps and outperforms previous methods.

## 1. Introduction

Person re-identification (ReID) aims to match images of the same individual captured by different cameras, playing an essential role in surveillance and intelligent monitoring systems. While most ReID methods focus on daytime RGB images, they face challenges in low-light or nighttime conditions. Visible–infrared person re-identification (VI-ReID) addresses this by matching visible (VIS) and infrared (IR) images, making ReID applicable in various lighting conditions. However, VI-ReID is more challenging due to significant modality gaps between VIS and IR images.

Two main methods have been developed to address this issue. The first is image-level methods [1,2,3,4], which utilize models like generative adversarial networks (GANs) to generate middle-modality features or new modality images. These methods, however, often encounter challenges such as unstable training, mode collapse, and issues with the generated samples, including color inconsistency and detail loss. These shortcomings negatively impact the performance of subsequent classification tasks. Recent studies have explored the use of diffusion models to generate new images [5]; however, they still fail to consistently produce high-quality images under real-world conditions. This indicates that relying solely on image-level methods is insufficient to bridge the modality gap. Another method is feature-level methods [6,7,8], which typically employ a two-step learning pipeline. Firstly, a backbone network extracts features from visible and infrared images, and then these features are projected into a common space to minimize the modality gap. Despite these efforts, the significant differences between modalities make direct mapping challenging. Both methods rely heavily on spatial information yet face difficulties in fully bridging the inherent modality gaps. Both image-level and feature-level methods have their merits, but their limitations in effectively addressing modality gaps underscore the need for a more comprehensive approach.

**Figure 1:**
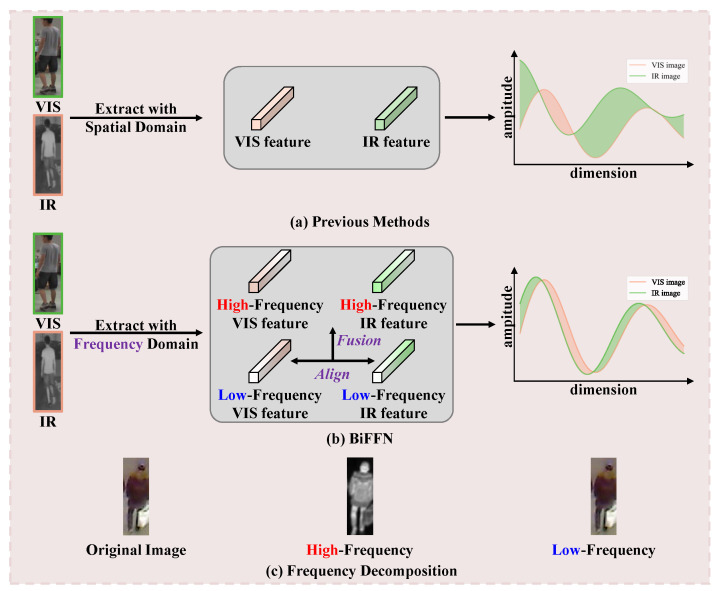
(**a**,**b**) Motivation of the proposed BiFFN, which aims to reduce modality gaps by extracting frequency domain features, aligning low-frequency features, and then fusing them with high-frequency features to generate middle-modality features. (**c**) Frequency decomposition: high-frequency images from wavelet transform capture rich textures, noise, and sharp edges, while low-frequency images contain stable global information like color and structure.

**Figure 2:**
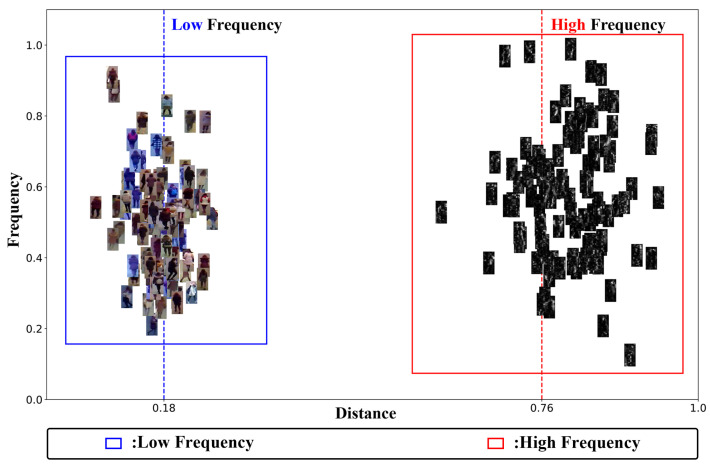
The distance distribution between high-frequency components, low-frequency images, and the original images on the RegDB dataset. The high-frequency components are farther from the original images, containing more discriminative information.

In recent years, frequency domain feature learning has shown great potential in various tasks such as image dehazing [9], image restoration [10], and low-light image enhancement [11]. Unlike spatial domain features, frequency information derives from the intrinsic physical properties of the image. Techniques such as wavelet transform are particularly effective in this context. As illustrated in Figure 1c, high-frequency images obtained through wavelet transform contain more texture details, noise, and sharper edge information. In contrast, low-frequency images capture stable global information, such as color and global structural information. **Specifically, low-frequency wavelets capture global content, whereas high-frequency wavelets capture edge details.** In Figure 2, we also illustrate the distances between the original image and its high- and low-frequency components. **There is a clear distinction between frequency and spatial-domain information, with the high-frequency components demonstrating more discriminative features.** Therefore, leveraging frequency-domain information and the strengths of both high- and low-frequency features is important for reducing modality gaps in VI-ReID tasks.

Building on this foundation, we propose a novel middle-modality feature generation method for VI-ReID, called the bi-frequency feature fusion network (BiFFN). **Our motivation is illustrated in Figure 1a,b**. BiFFN integrates high- and low-frequency domain features through three key modules: the frequency space enhancement (FSE) module, the deep frequency mining (DFM) module, and the cross-frequency fusion (CFF) module. Using discrete wavelet transform (DWT), high- and low-frequency features are extracted from the input. The FSE module has two branches: frequency enhancement, which uses high-frequency information to enhance low-frequency features, and spatial enhancement, which employs convolution to improve feature representation. The DFM module then processes these features to leverage edge information from the high-frequency domain and global structural information from the low-frequency domain. Finally, the CFF module aligns and fuses low- and high-frequency features, generating a middle-modality with comprehensive and effective information. To effectively align VIS, IR, and middle features, we introduce the uniform modality center (UMC) loss, which ensures balanced feature distances, promoting better alignment and integration of different modalities.

Our main contributions are as follows:

- We propose a novel bi-frequency feature fusion method that leverages the advantages of both high-frequency and low-frequency information to generate middle features for the VI-ReID task.
- We propose a DFM module that effectively extracts the advantageous information in high-frequency and low-frequency domains. Unlike previous methods that only consider the spatial domain, this novel method better utilizes the beneficial information inherent in both frequency domains. As a result, it significantly enhances feature representation and robustness.
- We propose a CFF module, combined with the UMC loss, to better align VIS, IR, and middle features in a common space, improving network training smoothness and preserving discriminative information.
- Extensive experiments demonstrate that our method significantly outperforms state-of-the-art methods across three datasets, highlighting its effectiveness.

## 2. Related Work

### 2.1. Visible–Infrared Person Re-Identification (VI-ReID)

Image-level VI-ReID methods reduce modality differences by converting images between VIS and IR. Some GAN-based approaches generate images that retain identity but vary in modal styles. However, the lack of paired VIS-IR images has made these generated images less reliable. Li et al. [1], Wei et al. [12], and Zhang et al. [13] proposed lightweight networks to generate middle modalities, which alleviated some modality gaps but still differed significantly from VIS/IR modalities. Feature-level methods aim to reduce modality gaps by mapping features into a common space. Some methods [14,15,16] have used CNN/VIT backbones for feature extraction. Zhang et al. [6] proposed a diversified embedding expansion network (DEEN) to learn feature information through diversified embeddings, while Qiu et al. [17] generated middle modalities by learning high-order structures. However, relying solely on spatial-domain information makes it challenging to properly distribute and map features in the common space. Unlike traditional feature-level methods, we introduce frequency-domain information in an innovative way and combine it with spatial-domain information to form a new feature representation. Traditional methods often struggle to generate sufficiently discriminative middle features from spatial-domain information alone. By incorporating frequency-domain data, and taking into account the beneficial information contained in both high- and low-frequency components, we apply distinct processing techniques to capture richer feature representations. When these features are fused with spatial information, the model becomes better at learning discriminative and robust features in the common space, overcoming the main limitations of existing approaches. Our method provides a novel and valuable contribution to the field of VI-ReID.

### 2.2. Frequency Domain Learning in VI-ReID

Frequency domain learning has found widespread application in the field of computer vision [18,19,20], particularly in cross-modality scenarios such as VI-ReID, where frequency domain representations can compensate for missing details in IR images captured under complex conditions. Numerous studies have demonstrated the effectiveness of frequency domain approaches in VI-ReID tasks. For example, Zhang et al. [21] introduced a Spatial-Frequency Token Selection (SFTS) module that adaptively selects object-centric tokens through both spatial and frequency information. In another work, Zhang et al. [22] proposed a Frequency Domain Nuances Mining (FDNM) method using Fourier transform to explore cross-modality frequency-domain information.

While these methods demonstrate promising results in utilizing frequency domain features, they neglect the inherent differences between frequency components and lack a global perspective for frequency-domain information, potentially limiting their ability to capture full-spectrum features essential for robust VI-ReID. To address these limitations, we propose the Bi-Frequency Feature Fusion Network (BiFFN), which integrates both high-frequency and low-frequency information to capture comprehensive discriminative features, thereby enabling more effective cross-modality feature extraction and alignment. This design overcomes the limitations of previous frequency domain-based methods that inadequately leverage both high- and low-frequency information in VI-ReID tasks. The combination of dual-frequency features in BiFFN enhances the discriminative power of learned representations, addressing the challenges of VI-ReID.

## 3. Methods

**Figure 3:**
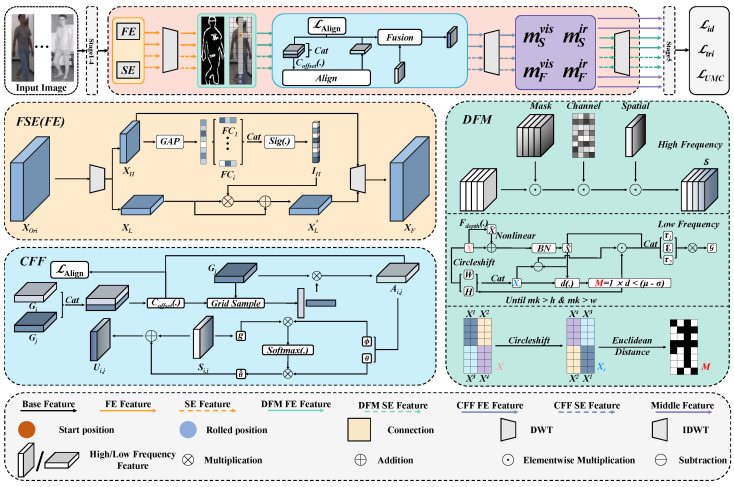
The proposed BiFFN network overview includes the following components: backbone, frequency-spatial enhancement (FSE) module, deep frequency mining (DFM) module, and cross-frequency fusion (CFF) module. These components are jointly optimized using the uniform modality center loss ($L_{\mathrm{UMC}}$), identity loss ($L_{\mathrm{id}}$), and triplet loss ($L_{\mathrm{tri}}$).

Figure 3 shows our proposed BiFFN architecture, built on the Attention Generalized mean pooling with Weighted triplet loss (AGW) [7] backbone and consisting of three key components: FSE, DFM, and CFF modules. The backbone processes VIS-IR image pairs, extracting features enhanced by the FSE module. The DFM module then extracts deep high- and low-frequency features, with high frequencies capturing semantic details and low frequencies extracting global information. The CFF module merges these into middle features, which are aligned and fused in a common space. The UMC loss is introduced to minimize the distances between VIS, IR, and middle features to enhance both the alignment and fusion of these features.

### 3.1. Frequency-Spatial Enhancement (FSE) Module

Traditional VI-ReID tasks have primarily focused on feature extraction using CNN or ViT, often overlooking the valuable information in the frequency domain. Our proposed FSE module, as shown in Figure 3, addresses this gap by enriching representations with diverse information from both frequency and spatial domains. This module effectively reduces the modality gaps between VIS and IR images. The FSE module has two branches: frequency enhancement (FE) and spatial enhancement (SE). The FE branch applies DWT to the input feature $X_{Ori}\in R^{C\times H\times W}$, splitting it into high-frequency $X_{H}$ and low-frequency features $X_{L}$, both of size $R^{C\times \frac{H}{2}\times \frac{W}{2}}$. High-frequency features are fed into global average pooling and then pass through *N* fully connected layers, forming *N*-dimensional vectors. These are concatenated, and their importance $I_{H}$ is calculated using the Sigmoid function Sig(·). High-frequency features enrich low-frequency features, resulting in enhanced low-frequency features $X_{L}^{\prime }$, expressed as follows:

(1) $$X_{L}^{\prime }=X_{L}+I_{H}⊗X_{L}.$$

The enhanced $X_{L}^{\prime }$ and $X_{H}$ are transformed back to the spatial domain using IDWT, producing the features $F_{F}^{vis}$ and $F_{F}^{ir}$ for the two modalities. The SE branch processes the input through three convolutional blocks, resulting in the features $F_{S}^{vis}$ and $F_{S}^{ir}$. The resulting feature set $X=\left\{F_{F}^{vis},F_{F}^{ir},F_{S}^{vis},F_{S}^{ir}\right\}$ serves as the input for the DFM module.

### 3.2. Deep Frequency Mining (DFM) Module

After extracting features with the FSE module, the network establishes dependencies between human features, low-frequency information, and spatial pixel regions. However, it does not fully exploit high- and low-frequency advantages. To address this, we designed the deep frequency mining (DFM) module (see Algorithm 1). The DFM module has two branches: human semantic mining (HSM) for high-frequency information and global structure learning (GSL) for low-frequency information. As shown in Figure 3, DFM uses DWT to decompose features into four sub-bands (L, H, V, D), where H, V, and D capture high-frequency edges and details, while L focuses on global information. H, V, and D are processed in the HSM branch, where mask and attention mechanisms aim to reduce background noise as much as possible while extracting relevant details. A learnable mask matrix $M_{H}$ reduces interference. This process provides channel attention weights $A_{\mathrm{channel}}$ and spatial attention weights $A_{\mathrm{spatial}}$ to refine the high-frequency information. The final output of the HSM branch is as follows:

(2) $$S=X_{M}⊙A_{\mathrm{channel}}⊙A_{\mathrm{spitial}}.$$

Low-frequency information contains richer details. Inspired by previous works [17,23], we designed the GSL branch to learn global structural features from various low-frequency components, enhancing feature representation. Instead of the computationally expensive k-nearest neighbor (KNN) used in traditional vision GNNs, we implement the graph relation matrix using a Dynamic Axial Graph. For the input feature *X*, a depthwise convolution is applied to each channel to embed positional information, resulting in $\tilde{X}$. After a nonlinear transformation, the feature representation is further enhanced through a Batch Normalization (BN) layer, resulting in $\hat{X}$. For the original feature map *X*, the operation is defined as follows:

(3) $$\begin{array}{cc} \; & X_{h,k}=Circleshift\left(X,k,\dim=2\right)\\ \; & X_{w,k}=Circleshift\left(X,k,\dim=3\right), \end{array}$$

where $Circleshift\left(X,k,\dim\right)$ denotes shifting the matrix *X* by *k* positions along the dim direction. The matrix is defined as follows:

(4) $$\begin{array}{cc} \; & H_{k}=Cat\left(X_{h,k}\left[:,:,:H/2,:\right],X_{h,k}\left[:,:,H/2:,:\right]\right)\\ \; & W_{k}=Cat\left(X_{w,k}\left[:,:,:,:W/2\right],X_{w,k}\left[:,:,:,W/2:\right]\right), \end{array}$$

where $W_{k}$ and $H_{k}$ represent the matrices obtained by shifting *X* by *k* positions vertically and horizontally, followed by flipping them vertically and horizontally, respectively. $Cat\left(\cdot \right)$ denotes the concatenation operation. The two matrices are concatenated along the channel dimension to obtain the feature map $X_{r}$. The standard deviation σ and mean μ of the Euclidean distance *D* between $\hat{X}$ and $X_{r}$ are calculated to construct the distance mask matrix *M* as follows:

(5) $$M_{i,j}=\left\{\begin{array}{l} 1,\;D_{i,j}<\mu -\sigma \\ 0,\;otherwise \end{array}\right..$$

The mask matrix *M* is applied to the difference set between $X_{r}$ and $\hat{X}$, followed by *m* steps of Circleshift until mk is greater than *H* and mk is greater than *W*. This process results in the masked-linked feature $X_{f}$. $X_{f}$ and $\hat{X}$ are then concatenated and multiplied with the linear transformation matrices $\tau_{1}$ and $\tau_{2}$, along with the nonlinear activation function ω, to form the graph structure *G*. This dynamic graph construction more effectively captures the structural features of the global information. Following the above process, the DFM module obtains two feature sets, S and G, through the HSM and GSL branches. Each branch deeply mines information from the high-frequency and low-frequency components, respectively, i.e.,

(6) $$\begin{array}{cc} \; & S=\left\{S_{S}^{vis},S_{S}^{ir},S_{F}^{vis},S_{F}^{ir}\right\},G=\left\{G_{G}^{vis},G_{S}^{ir},G_{F}^{vis},G_{F}^{ir}\right\}. \end{array}$$

**Algorithm 1** Pseudo code of the DFM module1:**Input:** Feature map $X\in R^{C\times H\times W}$2:**Output:** High-frequency features *S*, Low-frequency graph *G*3: 4:**Step 1: Frequency Decomposition**5:L,H,V,D←DWT(X)                   ▹ Wavelet Decomposition6: 7:**Step 2: High-frequency Mining (HSM)**8:$X_{\mathrm{high}}\leftarrow \mathrm{Concat}\left(H,V,D\right)$9:$M\leftarrow \mathrm{LearnableMask}(X_{\mathrm{high}})$10:$A_{c}\leftarrow \mathrm{ChannelAttention}\left(X_{\mathrm{high}}⊙M\right)$11:$A_{s}\leftarrow \mathrm{SpatialAttention}\left(X_{\mathrm{high}}⊙M\right)$12:$S\leftarrow X_{\mathrm{high}}⊙M⊙A_{c}⊙A_{s}$13: 14:**Step 3: Low-frequency Learning (GSL)**15:$\hat{X}\leftarrow \mathrm{BN}\left(\sigma \left(\mathrm{DepthwiseConv}\left(L\right)\right)\right)$                   ▹σ=ReLU16:Initialize $X_{f}=0$17:**for** k=1 **to** *m* **do**18:    **Dynamic Axial Graph Construction (Formula (3))**19:    $X_{hk}\leftarrow Circleshift\left(\hat{X},k,\dim=H\right)$20:    $H_{k}\leftarrow \mathrm{Concat}\left(X_{hk}\left[:,:H/2\right],\mathrm{Flip}\left(X_{hk}\left[:,H/2:\right]\right)\right)$21:    $X_{wk}\leftarrow Circleshift\left(\hat{X},k,\dim=W\right)$22:    $W_{k}\leftarrow \mathrm{Concat}\left(X_{wk}\left[:,:,:W/2\right],\mathrm{Flip}\left(X_{wk}\left[:,W/2:\right]\right)\right)$23:    $X_{r}\leftarrow \mathrm{Concat}\left(H_{k},W_{k}\right)$24:    **Adaptive Mask Calculation (Formula (4))**25:    $D\leftarrow \mathrm{EuclideanDistance}(\hat{X},X_{r})$26:    μ,σ←Mean(D),Std(D)27:    $M\leftarrow I_{D<\mu -\sigma }$28:    $X_{f}\leftarrow X_{f}+M⊙\left(X_{r}-\hat{X}\right)$29:    **if** k>max(H,W)/m **then**30:        **break**31:    **end if**32:**end for**33:**Graph Structure Generation**34:$G\leftarrow \tau_{2}\left(\sigma \left(\tau_{1}\left(\mathrm{Concat}\left(X_{f},\hat{X}\right)\right)\right)\right)$        ▹τ represents linear transformations35:**return** S,G

### 3.3. Cross-Frequency Fusion (CFF) Module

The features obtained through the DFM module are processed through a deeper extraction in both the high-frequency and low-frequency domains. However, to fully utilize the effective information from both domains, we need to generate middle features that align them in a common space. Therefore, we use the CFF module to further extract and fuse the effective information from both feature sets, generating more representative middle features. For the feature set G, we obtain its graph structure with global structural features in the DFM module. Based on this, we align each feature in the feature set G with the other three. We define an offset *O* to represent the distance between the features in space. Taking $G_{F}^{vis}$ as an example, its offset with $G_{S}^{vis}$ is defined as follows:

(7) $$O_{F}^{vis}=C_{\mathrm{offset}}\left(Cat\left(G_{F}^{vis},G_{S}^{vis}\right)\right),$$

where $C_{\mathrm{offset}}$ denotes the operation of calculating the offset by convolution. Based on this offset, a sampling matrix weighted by the distance is derived to generate the aligned features, i.e.,

(8) $$\begin{array}{c} A_{S,F}^{vis,vis}=G_{F}^{vis}⊗GridSample\left(G_{F}^{vis},O_{F}^{vis}\right), \end{array}$$

where $GridSample\left(\cdot \right)$ denotes the gridsample operator. To encourage the model to better align the two features, we use the following formula to calculate the alignment loss $L_{\mathrm{Align}}$:

(9) $$L_{\mathrm{Align}}\left(A_{S,F}^{vis,vis}\right)=\frac{1}{N_{A}}\sum_{i=1}^{N_{A}}{\left(O_{F_{i}}^{vis}-A_{S,F_{i}}^{vis,vis}\right)}^{2},$$

where $N_{A}$ represents the total number of elements; $O_{F_{i}}^{vis}$ and $A_{S,F_{i}}^{vis,vis}$ denote the values of the *i*th element in $O_{F}^{vis}$ and $A_{S,F}^{vis,vis}$, respectively. This formula calculates the mean squared difference between corresponding positions in the feature maps $O_{F}^{vis}$ and $A_{S,F}^{vis,vis}$ as the alignment loss. As shown in Equation (8), aligning $G_{S}^{vis}$ with $G_{S}^{ir}$, $G_{F}^{vis}$, and $G_{F}^{ir}$ results in the aligned low-frequency feature $A_{S}^{vis}$. Similarly, we obtain the aligned low-frequency feature set $A=\left\{A_{S}^{vis},A_{S}^{ir},A_{F}^{vis},A_{F}^{ir}\right\}$ and fuse it with high-frequency features. For example, linear transformations of $S_{F}^{vis}$ and $A_{S}^{vis}$ produce $g_{F}^{vis}$, $\phi_{S}^{vis}$, and $\theta_{S}^{vis}$. The correlation matrix $E=g_{F}^{vis}⊗\phi_{S}^{⊤vis}$ is normalized to obtain $E^{\prime }$, which is then weighted, summed with $\theta_{S}^{vis}$, and further processed to obtain $\hat{\theta }$. Adding $\hat{\theta }$ to $S_{F}^{vis}$ results in the fused high-frequency feature $U_{F,S}^{vis,vis}$. Following the same procedure, we obtain the fused high-frequency set U. Then, the fused high-frequency set U and low-frequency set A are then restored to the spatial domain using IDWT, generating the middle modality feature set M. Each feature in $M=\left\{M_{S}^{vis},M_{S}^{ir},M_{F}^{vis},M_{F}^{ir}\right\}$ is processed through global and partial generalized mean pooling, and the pooled features are concatenated to obtain the 1D middle feature $M^{\prime }=\left\{m_{SE}^{vis},m_{SE}^{ir},m_{F}^{vis},m_{F}^{ir}\right\}$. Similarly, the middle feature $D^{\prime }=\left\{d_{SE}^{vis},d_{SE}^{ir},d_{F}^{vis},d_{F}^{ir}\right\}$ is derived from S and G. To further reduce intra-class and inter-class gaps in the middle features, we propose the uniform modality center (UMC) loss. This loss minimizes the distance between VIS, IR, and middle modalities while preserving discriminative identity information. VI-ReID aims to reduce the distance between the same identity across modalities while maintaining the distinction between different identities. Our loss function contains two components: identity difference loss and triple center loss.

**Figure 4:**
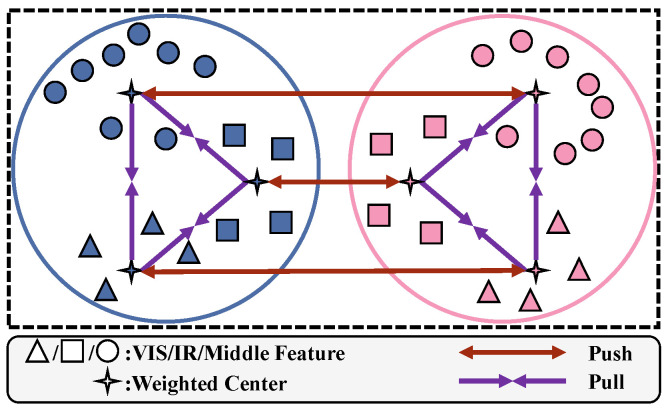
Illustration of the proposed UMC loss. Different colors represent different identities.

As shown in Figure 4, we first calculate the weighted center for each identity and modality. Given the feature vector set $\left\{f^{l_{1},m_{1}},f^{l_{2},m_{2}},\ldots ,f^{l_{q},m_{n}}\right\}$ for label *l* and modality *m*, the weighted center $c^{lm}$ can be expressed as follows:

(10) $$\begin{array}{c} c^{l,m}=\sum_{i}\left(\frac{\exp\left(\sum_{j}\frac{f_{i}^{l,m}\cdot f_{j}^{l,m}}{∥f_{i}^{l,m}∥∥f_{j}^{l,m}∥}\right)}{\sum_{k}\exp\left(\sum_{j}\frac{f_{k}^{l,m}\cdot f_{j}^{l,m}}{∥f_{k}^{l,m}∥∥f_{j}^{l,m}∥}\right)}\right)f_{i}^{l,m}, \end{array}$$

where $f_{i}^{l,m}$ represents the *i*th feature vector for identity *l* and modality *m*. By calculating these, we obtain the weighted center set $c^{l,m}$ for all labels $l_{q,\;q\in \{1,\ldots ,q\}},$ and all modalities set $m_{n,\;n\in \{vis,ir,inter\}}$. The purpose of the identity difference loss is to pull the center distances of the same identity closer while pushing the center distances of different identities further apart. Given the modality center set $\left\{c^{l,m_{vis}},c^{l,m_{ir}},c^{l,m_{mid}}\right\}$ for label *l* and the modality center set $\left\{c^{l^{\prime },m_{vis}},c^{l^{\prime },m_{ir}},c^{l^{\prime },m_{mid}}\right\}$ for label $l^{\prime }$, the identity difference loss can be expressed as follows:

(11) $$\begin{array}{cc} L_{IdD} & =\sum_{l}\sum_{i<j}{∥c_{i}^{lm}-c_{j}^{lm}∥}_{2}\\ \; & +\sum_{l\neq l^{\prime }}\sum_{m}\max(0,\alpha -∥c^{lm}-c^{l^{\prime }m}{∥}_{2}), \end{array}$$

where α is a hyperparameter used to control the minimum distance between the centers of different identities within the same modality. The inter-class distances among VIS, IR, and the middle modality (mid) are constrained using the triple center loss as shown below:

(12) $$\begin{array}{cc} L_{triCenter}=\sum_{l}( & ∥c^{l,m_{vis}}-c^{l,m_{ir}}{∥}_{2}\\ + & ∥c^{l,m_{vis}}-c^{l,m_{mid}}{∥}_{2}\\ + & ∥c^{l,m_{ir}}-c^{l,m_{mid}}{∥}_{2})/3. \end{array}$$

Therefore, our UMC loss can be expressed as follows:

(13) $$\begin{array}{c} L_{UMC}=L_{\mathrm{Align}}+L_{IdD}+L_{triCenter}. \end{array}$$

### 3.4. Multi-Loss Optimization

In addition to $L_{UMC}$, we also use the cross-entropy loss $L_{id}$ and the triplet loss $L_{tri}$ [24] to jointly optimize the proposed BiFFN. Therefore, the final loss can be expressed as follows:

(14) $$\begin{array}{c} L=L_{UMC}+L_{id}+L_{tri}. \end{array}$$

## 4. Experiment and Analysis

### 4.1. Datasets

The SYSU-MM01 dataset [25] consists of 491 identities, with photos captured by 4 VIS and 2 IR cameras, totaling 30,071 VIS images and 15,792 IR images. It includes two modes: All Search and Indoor Search. In All Search, all images captured by the VIS cameras are used as the gallery set, whereas in Indoor Search, only images captured by the two indoor VIS cameras are used as the gallery set. The RegDB dataset [26] includes 412 identities, each with ten pairs of VIS-IR images captured by overlapping cameras. The LLCM dataset [6] captures images in low-light environments. Its training set includes 713 identities, with 16,946 VIS images and 13,975 IR images, while the test set contains 351 identities, with 8680 VIS images and 7166 IR images.

### 4.2. Implementation Details

All images are resized to 3 × 288 × 160 before being fed into the network. We randomly selected 8 identities for training in each mini-batch, with 4 VIS images and 4 IR images for each identity. We used AGW [7] as our backbone. The initial learning rate was set to $1\times 10^{-2}$, warmed up to $1\times 10^{-1}$ in the first 10 epochs, decayed to $1\times 10^{-2}$ at the 20th epoch, and further decayed to $1\times 10^{-3}$ and $1\times 10^{-4}$ at the 60th and 120th epochs, respectively, for a total of 150 epochs. Training employs Stochastic Gradient Descent (SGD) as the optimizer with a momentum parameter 0.9. Our proposed BiFFN was implemented using PyTorch 1.8 on an RTX 4090 GPU (NVIDIA Corporation, Santa Clara, CA, USA).

We evaluated the performance using Cumulative Matching Characteristic (CMC) and mean Average Precision (mAP). CMC assesses the probability of a true match being ranked within the top-k retrieval results. Following the practice of HOS-Net [17], for the RegDB dataset, we performed a random split for training and testing, repeating the process ten times to obtain the average performance. Likewise, for the SYSU-MM01 and LLCM datasets, we randomly split the gallery set ten times and report the average performance.

### 4.3. Comparison with State-of-the-Art Methods

To demonstrate the effectiveness of BiFFN, we compared it with several state-of-the-art or most representative methods, as shown in Table 1.

**SYSU-MM01.** As shown in Table 1, our proposed BiFFN outperforms all the compared methods. Specifically, under the Indoor Search mode, BiFFN achieves a Rank-1 accuracy of 85.8% and an mAP of 87.9%. It also performs excellently under the All Search mode, with a Rank-1 accuracy of 77.5% and an mAP of 75.9%. Our method demonstrates superior performance when compared to both image-level methods and feature-level methods. This indicates that, under well-lit conditions, the network we designed effectively reduces the modality gap, leading to excellent performance on the SYSU-MM01 dataset.
**RegDB.** From Table 1, it can be observed that our proposed BiFFN achieves the best performance in both search modes. Under the two search modes, our BiFFN outperforms DEEN by 3.8%/7.8% and 4.6%/8.3% in Rank-1/mAP, respectively. Compared with the second-best method, HOS-Net, our BiFFN also shows a 2.5% improvement in mAP in both modes. Both our BiFFN and HOS-Net achieve impressive results on the RegDB dataset, showing a significant performance gap compared to other methods. Considering the network architecture, both our method and HOS-Net incorporate graph-based structures, which allow for richer feature representations of the targets. However, since our BiFFN also leverages frequency-domain information, it outperforms HOS-Net, resulting in better overall performance.
**LLCM.** We also report experimental results on the LLCM dataset, as shown in Table 1. The LLCM dataset is designed to better simulate real-world VI-ReID scenarios by capturing pedestrian images in low-light environments, which typically have higher noise levels. Methods relying solely on spatial-domain information struggle on this dataset. Our proposed BiFFN, which incorporates frequency-domain information and reduces dependence on spatial domain features, performs exceptionally well. Compared to the second-best method, HOS-Net, our Rank-1 is improved by 3.7% under the IR to VIS mode and by 3.0%/1.0% in Rank-1/mAP under the VIS to IR mode. Although the LLCM dataset is relatively new with limited reference methods, our approach still shows significant improvements. These results indicate that the frequency-domain information in our method effectively handles more challenging VI-ReID tasks.

### 4.4. Ablation Studies

#### 4.4.1. Effectiveness of Key Components

To evaluate each component’s contribution to BiFFN, we conducted ablation studies on the SYSU-MM01 and LLCM datasets by adding key components to the baseline and assessing their influence on performance. The network setup remained unchanged, with only the tested modules added to or removed from BiFFN. The results are shown in Table 2.

**FSE:** Compared to the baseline, adding the FSE module improved Rank-1/mAP by 5.0% and 5.8%, respectively, on the SYSU-MM01 dataset. Notably, on the LLCM dataset, the FSE module significantly enhanced performance, with Rank-1/mAP increasing by 9.0% and 5.3%, respectively. This demonstrates that the FSE module effectively enhances the network’s robustness by introducing frequency-domain information in low-light and noisy environments. **DFM:** The DFM module boosted Rank-1/mAP by 14.3%/6.7% on SYSU-MM01 and 6.2%/8.5% on LLCM compared to the baseline. When combined with the FSE module, the mAP on SYSU-MM01 increased by an additional 4.5%, highlighting the DFM module’s ability to mine and enhance both high and low-frequency features. **CFF and $L_{UMC}$:** Adding the CFF module improved Rank-1/mAP by 2.3%/1.3% on LLCM, demonstrating its effectiveness in feature alignment and fusion. However, the most significant improvements came after incorporating the $L_{UMC}$ loss, which increased mAP by 2.7% on SYSU-MM01 and 0.6% on LLCM, while improving Rank-1 by 1.5% on LLCM. This indicates that $L_{UMC}$ effectively enhances the CFF module, enabling BiFFN to perform better in the VI-ReID task.

#### 4.4.2. Influence of Different High–Low Feature Combination Methods

The CFF module generates middle features for classification by aligning low-frequency components and fusing them with high-frequency ones. Table 3 compares the performance of different combination methods. Addition simply combines the features by summing them element-wise. However, this method may fail to capture higher-order interactions between features, especially when they vary significantly in scale or importance. Moreover, it can lead to information loss when one set of features dominates the other. In contrast, the CFF module first aligns the low-frequency features with the high-frequency ones, allowing for a more controlled integration of both features. This alignment reduces potential conflicts between the features and ensures that they complement each other. When fused, the features maintain their distinct characteristics while enhancing the model’s ability to differentiate between various classes, thus avoiding the downsides of traditional methods. Our fusion + align approach outperforms addition, concatenation, and fusion or alignment alone on the LLCM dataset, indicating the effectiveness of the CFF module’s method.

#### 4.4.3. Effectiveness of Bi-Frequency and Corresponding Methods

BiFFN reduces modality gaps by applying different methods to high-frequency (HF) and low-frequency (LF) features. We conducted studies by selecting different frequency features and transferring the methods (e.g., in the DFM module, high-frequency features should correspond to the HSM branch, while low-frequency features should correspond to the GSL branch. However, after transformation, high-frequency features were matched with the GSL branch, and low-frequency features were matched with the HSM branch. Additionally, we also explored the approach of retaining only a single frequency branch and leaving the other branch unprocessed.) applied to these features while keeping other network settings unchanged. The results, presented in Table 4, show that using individual frequency features leads to performance improvement; however, it falls short compared to the dual-frequency approach. This validates the effectiveness of utilizing dual frequencies in our method. Additionally, when the methods applied to the respective frequencies were transferred, there was a noticeable performance decline, indicating that the methods chosen for each frequency in BiFFN are both appropriate and effective.

#### 4.4.4. Effectiveness of Each Term in $L_{UMC}$

The UMC loss is proposed to reduce modality gaps while preserving discriminative identity information. As shown in Table 5, each term in the UMC loss contributes to improving the performance of BiFFN during training. When all terms in the UMC loss are used to optimize the network, the Rank-1/mAP improves by 1.6%/0.7% and 1.2%/2.8% compared to BiFFN without UMC loss optimization. This indicates that the UMC loss effectively reduces modality gaps, leading to more well-distributed features in the feature space.

#### 4.4.5. Effectiveness of Dynamic Axial Graph

The DFM module’s GSL branch models the global structure using a dynamic axial graph. We replaced it with the hypergraph network from HOS-Net and the SVGA from MobileViG, and compared their performance and parameter count on the LLCM dataset. As shown in Table 6, our Dynamic Axial Graph achieves the best performance with the fewest parameters. The static graph construction in SVGA fails to learn global structural features effectively. Incorporating the Dynamic Axial Graph improved R-1/mAP by 7.3%/2.6%, demonstrating its effectiveness in reducing parameters and enhancing global feature learning through dynamic construction. Unlike HyperGraph, which requires many connections and, thus, increases computational complexity, and SVGA, which constructs fixed network relationships and lacks flexibility, the Dynamic Axial Graph achieves superior performance with reduced computational costs. This makes it more suitable for the VI-ReID task, where efficiency and adaptability are critical.

#### 4.4.6. Influence of Different Hyperparameters α and *k*

**Figure 5:**
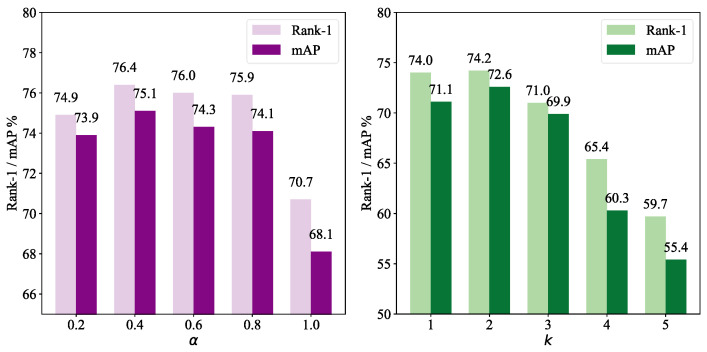
The influence of different hyperparameters α and *k*.

To evaluate the influence of the two hyperparameters on performance, we conducted a quantitative comparison, and the results are shown in Figure 5. For the hyperparameter *k*, considering the size of the feature map, we set its maximum value to 5 in the experiments. We observed that BiFFN performed best when k=2 and α=0.4.

#### 4.4.7. Influence of the Stage of the Backbone on Plugging the Key Components

Our key components (FSE, DFM, CFF) can be plugged into any stage of the backbone network. In this study, we use AGW as the backbone, which consists of five stages. We plugged the key components into different stages of the backbone to evaluate their performance. As shown in Table 7, the best performance was achieved when the key components were plugged in after stage 4. This indicates that the high- and low-frequency information extracted at stage 4 is particularly beneficial for our key components to enhance the model’s learning, resulting in more well-distributed middle modality features.

### 4.5. Visualization Analysis

To further verify the effectiveness of BiFFN, we performed visualizations on the SYSU-MM01 and RegDB datasets.

#### 4.5.1. Intra-Class and Inter-Class Distances

**Figure 6:**
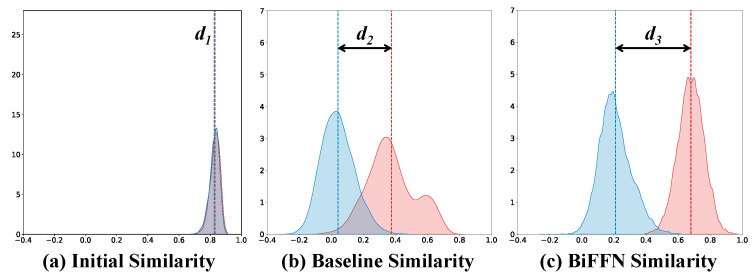
(**a**–**c**) The distributions of intra-class and inter-class similarities of VIS and IR modality features on the RegDB dataset.

As shown in Figure 6a–c, compared to the initial feature distances and baseline feature distances, our proposed BiFFN significantly separates the inter-class distances and intra-class distances (d3>d2>d1≈0). This indicates that our method can effectively reduce the modality gap, achieving better performance.

#### 4.5.2. Feature Distribution

**Figure 7:**
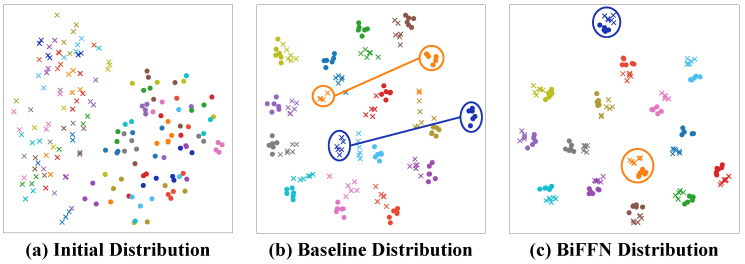
(**a**–**c**) The t-SNE distribution of the VIS-IR features. Different colors represent different identities. The “circle” and “cross” markers represent the VIS and IR features, respectively.

We also visualized the feature distribution in space using t-SNE [35]. As shown in Figure 7a–c, our method achieves a more reasonable spatial distribution compared to the initial and baseline spatial distributions, further demonstrating our approach’s effectiveness for the VI-ReID task.

#### 4.5.3. Retrieval Results

**Figure 8:**
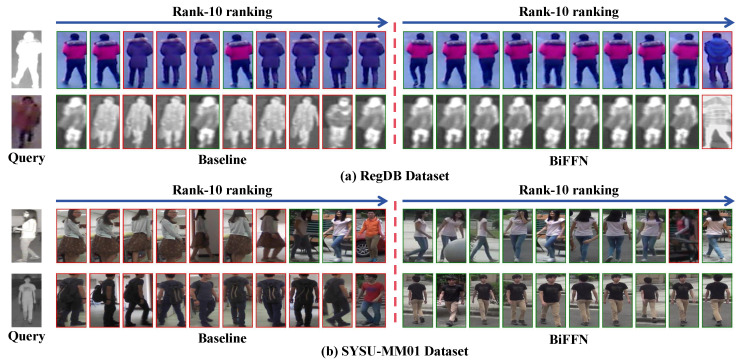
(**a**,**b**) The Rank-10 retrieval results obtained by the baseline method and the proposed BiFFN method on the SYSU-MM01 and RegDB datasets.

To visually demonstrate the excellent performance of our proposed BiFFN, we present retrieval results on the SYSU-MM01 and RegDB datasets in Figure 8a,b. Green boxes represent correct matches, while red boxes represent incorrect matches. Overall, our method significantly improves the ranking results, more accurately matching the correct identities in retrieval tasks.

#### 4.5.4. Heatmap

**Figure 9:**
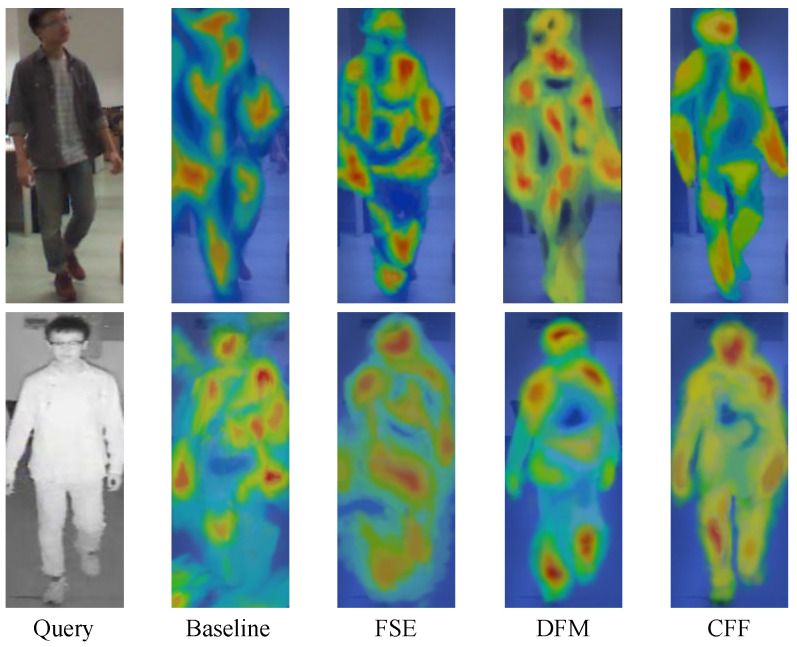
Heatmap visualization of each module on the SYSU-MM01 dataset.

To further demonstrate the impact of each module on the network, we performed heatmap visualizations on the network after incorporating the modules in Figure 9. Both for VIS and IR images, the network with the FSE module applied showed improved feature extraction for the person, with a stronger focus on the subject compared to the baseline. After further processing through the DFM module, the VIS image exhibited regional attention to the global structure of the body. For the IR image, although less pronounced than in the VIS image, there was still an enhanced treatment of the body’s global structure. However, in the DFM module, the VIS image showed some over-attention to certain areas. This issue was resolved after the CFF module was applied, as the final heatmaps, both for VIS and IR images, displayed a more balanced focus on the human body, with a well-controlled attention scope. This visualization further clarifies the role of each module in the network and its overall effectiveness.

## 5. Conclusions

In this paper, we propose a novel BiFFN that enhances feature representation by leveraging information from both high- and low-frequency domains to generate middle features, effectively reducing modality gaps. The FSE module strengthens low-frequency feature representation by integrating critical high-frequency information with spatial domain data. The DFM module extracts the strengths of both high- and low-frequency features, while the CFF module aligns and fuses these features to produce middle features, significantly minimizing modality gaps. Extensive experiments on three VI-ReID benchmark datasets confirm the effectiveness of BiFFN against state-of-the-art methods.

## Figures and Tables

**Table 1:** Comparison with state-of-the-art methods on the SYSU-MM01, RegDB, and LLCM datasets. Bold and underline highlight the best and second-best performance, respectively.

Methods	SYSU-MM01		RegDB		LLCM	
	**All-Search**	**Indoor-Search**	**VIS to IR**	**IR to VIS**	**VIS to IR**	**IR to VIS**
	**R-1/mAP**	**R-1/mAP**	**R-1/mAP**	**R-1/mAP**	**R-1/mAP**	**R-1/mAP**
$D^{2}L$ [27]	28.9/29.2	-/-	43.4/44.1	-/-	-/-	-/-
Align-GAN [3]	42.4/40.7	45.9/54.3	57.9/53.6	56.3/53.4	-/-	-/-
X-Modality [1]	49.9/50.7	-/-	62.2/60.2	-/-	-/-	-/-
DDAG [8]	54.8/53.0	61.0/68.0	69.3/63.5	68.1/61.8	48.0/52.3	40.3/48.4
LbA [28]	55.4/54.1	58.5/66.3	74.2/67.6	67.5/72.4	50.8/55.6	43.8/53.1
$G^{2}\mathrm{DA}$ [29]	63.9/60.7	71.0/76.0	74.0/65.5	69.7/62.0	-/-	-/-
FMCNet [30]	66.3/62.5	68.2/74.1	89.1/84.4	88.4/83.9	-/-	-/-
PMT [16]	67.5/65.0	71.7/76.5	84.8/76.6	84.2/75.1	-/-	-/-
CAJ [7]	69.9/66.9	76.3/80.4	85.0/79.1	84.8/77.8	56.5/59.8	48.8/56.6
MMN [13]	70.6/66.9	76.2/79.6	91.6/84.1	87.5/80.5	59.9/62.7	52.5/58.9
MAUM [31]	71.7/68.8	77.0/81.9	87.9/85.1	87.0/84.3	-/-	-/-
DEEN [6]	74.7/71.8	80.3/83.3	91.1/85.1	89.5/83.4	62.5/65.8	54.9/62.9
MSCLNet [32]	77.0/71.6	78.5/81.2	84.2/81.0	83.9/78.3	-/-	-/-
SGIEL [33]	77.1/72.3	82.1/83.0	92.2/86.6	91.1/85.2	-/-	-/-
HOS-Net [17]	75.6/74.2	84.2/86.7	94.7/90.4	93.3/89.2	64.9/67.9	56.4/63.2
MSCMNet [34]	**78.5**/74.2	83.0/85.5	90.4/81.2	87.7/78.2	63.9/66.1	55.1/60.8
**BiFFN (Ours)**	77.5/**75.9**	**85.8**/**87.9**	**94.9**/**92.9**	**94.1**/**91.7**	**66.8**/**68.6**	**58.5**/**63.7**

**Table 2:** Effectiveness of bi-frequency and corresponding methods in VI-ReID. Bold values indicate the best performance.

Settings	LLCM		SYSU-MM01	
	**Rank-1**	**mAP**	**Rank-1**	**mAP**
baseline	48.8	56.6	69.9	66.9
baseline+FSE	53.2	59.6	73.4	70.8
baseline+DFM	55.8	60.4	74.2	72.6
baseline+FSE+DFM	56.6	60.7	74.5	74.0
baseline+DFM+CFF	57.1	61.2	75.2	74.6
baseline+DFM+CFF+$L_{UMC}$	57.8	62.9	76.4	75.1
baseline+FSE+DFM+CFF+$L_{UMC}$	**58.5**	**63.7**	**77.5**	**75.9**

**Table 3:** The influence of each component on the performance of the proposed BiFFN on the LLCM and SYSU–MM01 datasets. Bold values indicate the best performance.

Methods	LLCM		SYSU-MM01	
	**Rank-1**	**mAP**	**Rank-1**	**mAP**
Addition	53.9	61.3	74.8	74.5
Concatenation	52.9	55.9	74.1	73.2
Align	56.9	61.5	75.2	73.7
Fusion	57.2	60.4	75.3	73.4
Align + Fusion	**57.8**	**62.9**	**76.4**	**75.1**

**Table 4:** Effectiveness of bi-frequency and corresponding methods. Bold values indicate the best performance and ✔ indicates the method is selected.

Component	HF	LF	Method Transfer	Rank-1	mAP
FSE	✔	✔	✔	70.2	68.1
	✔	✔		**73.4**	**70.8**
DFM	✔			72.1	70.6
	✔		✔	69.3	65.9
		✔		72.6	70.9
		✔	✔	69.0	65.4
	✔	✔	✔	69.4	65.9
	✔	✔		**74.2**	**72.6**
CFF	✔			75.1	73.2
	✔		✔	73.3	71.8
		✔		75.4	73.8
		✔	✔	72.0	71.2
	✔	✔	✔	72.9	70.5
	✔	✔		**76.4**	**75.1**

**Table 5:** Effectiveness of different combinations of IDD, TRIC, and alignment. Bold values indicate the best performance.

Settings	SYSU-MM01		LLCM	
	**Rank-1**	**mAP**	**Rank-1**	**mAP**
-	75.2	74.6	57.1	61.2
$+L_{IdD}$	75.4	74.9	57.5	61.8
$+L_{triC}$	75.3	74.9	57.4	62.2
$+L_{IdD}+L_{triC}$	76.0	75.1	57.7	62.4
$+L_{IdD}+L_{triC}+L_{\mathrm{Align}}$	**76.4**	**75.1**	**57.8**	**62.9**

**Table 6:** The influence of different graph constructions on the LLCM dataset. Bold values indicate the best performance or the smallest number of parameters.

Methods	Parameters	Rank-1	mAP
baseline	-	53.2	59.6
SVGA	14.2M	49.0	50.4
HyperGraph	14.1M	54.4	59.6
Dynamic Axial Graph	**12.7M**	**57.1**	**61.2**

**Table 7:** The influence of the stage of backbone to plug the FSE, DFM, and CFF. Bold values indicate the best performance.

Settings	SYSU-MM01		LLCM	
	**Rank-1**	**mAP**	**Rank-1**	**mAP**
baseline	69.9	66.9	48.8	56.6
plugged after stage 1	71.0	67.0	50.5	56.7
plugged after stage 2	72.1	67.6	50.8	56.4
plugged after stage 3	72.4	69.2	51.7	56.9
plugged after stage 4	**77.5**	**75.9**	**58.6**	**63.7**
plugged after stage 5	74.2	72.3	53.5	58.1

## Data Availability

The data that support the findings of this study are available online. These datasets were derived from the following public resources: [SYSU-MM01, RegDB, LLCM].
